# Oxidative Stress in Neonatal Disease: Established Organ Injury and Emerging Evidence for Skeletal Involvement

**DOI:** 10.1155/omcl/8655644

**Published:** 2026-10-09

**Authors:** Laura Cannavò, Virginia Beretta, Chiara Petrolini, Vincenzo Raitano, Elena Scarpa, Valentina Dell’Orto, Silvia Carloni, Maria Cristina Albertini, Serafina Perrone

**Affiliations:** ^1^ Pediatric Intensive Care Unit University Hospital of Verona Piazzale Stefani 1 Verona 37126 Italy univr.it; ^2^ Neonatology Unit Department of Medicine and Surgery University of Parma Pietro Barilla Children’s Hospital Parma 43126 Italy unipr.it; ^3^ Department of Biomolecular Sciences University of Urbino Carlo Bo Urbino Italy uniurb.it

**Keywords:** antioxidant strategies, biomarkers, free radicals, newborns, oxidative stress, skeletal development

## Abstract

Reactive oxygen and nitrogen species participate in physiological signaling and adaptation during fetal and neonatal development, whereas excessive, prolonged, or spatially dysregulated production may contribute to oxidative and nitrosative tissue injury. Redox dysregulation has been extensively investigated in neonatal conditions, including bronchopulmonary dysplasia (BPD), hypoxic‐ischemic encephalopathy, retinopathy of prematurity (ROP), necrotizing enterocolitis (NEC), congenital heart disease (CHD), and hyperbilirubinemia. Its relationship with bone metabolism is well established in adult and pediatric disorders, including osteoporosis, inflammatory bone loss, and iron‐overload‐related bone disease. However, the potential contribution of perinatal redox imbalance to early skeletal development remains comparatively underexplored. This narrative review critically examines the biochemical mechanisms of reactive species generation, their interaction with inflammatory pathways, and the experimental and clinical evidence linking redox dysregulation to neonatal organ injury. Particular attention is given to the developing skeleton as a potentially underrecognized target. Experimental studies indicate that excessive reactive species can impair chondrogenesis, alter osteoblast and osteoclast activity, disrupt collagen and osteogenic signaling, and interfere with skeletal growth. Human evidence is predominantly observational. Preliminary longitudinal data show inverse associations between cord‐blood markers of oxidative DNA and protein modification and REMS‐derived skeletal Z‐scores during the first year of life. These findings suggest that early redox imbalance may impair bone quality and growth trajectories in newborns. Prospective studies integrating standardized redox biomarkers, bone‐metabolism indices, imaging, nutrition, medication exposure, growth, and fracture outcomes are needed to determine whether redox dysregulation independently contributes to impaired skeletal development.

## 1. Introduction

Free radicals (FRs) and other reactive species are continuously generated during the normal cellular metabolism. Rather than being intrinsically harmful, reactive oxygen species (ROS) and reactive nitrogen species (RNS) act as signaling mediators when maintained within a physiological range [1]. Their biological effects depend on their concentration, chemical identity, subcellular localization, duration of exposure, and the developmental and metabolic context in which they are produced. At controlled levels, ROS and RNS contribute to the regulation of vascular tone, host defense, oxygen sensing, mitochondrial adaptation, gene expression, cell proliferation and differentiation, and tissue development [2, 3]. Accordingly, redox homeostasis should not be understood as the complete elimination of reactive species but as the maintenance of spatially and temporally regulated redox signaling.

Redox homeostasis is supported by an integrated antioxidant network that includes enzymatic systems, such as superoxide dismutases, catalase, glutathione peroxidases, peroxiredoxins, and thioredoxin‐dependent pathways, as well as non‐enzymatic antioxidants, including vitamins C and E [4, 5]. These systems do not merely neutralize reactive species; they also regulate redox‐sensitive signaling and limit the propagation of oxidative reactions.

The transition from intrauterine to extrauterine life is accompanied by a marked increase in oxygen exposure and profound metabolic adaptation. Consequently, the neonatal period is characterized by dynamic changes in redox signaling rather than by oxidative stress (OS) per se [6]. Oxidative or nitrosative stress arises when reactive species production becomes excessive, prolonged, or spatially dysregulated relative to antioxidant and repair capacity. This balance is particularly vulnerable in preterm infants because antioxidant systems, placental transfer of antioxidant compounds, and endogenous antioxidant reserves may be incompletely developed, while exposure to supplemental oxygen, inflammation, infection, parenteral nutrition, and intensive care interventions may increase the oxidative burden [7, 8].

When redox regulation is disrupted, reactive species may induce or amplify damage to lipids, proteins, nucleic acids, mitochondria, and cellular membranes [9]. However, their mechanistic role is context‐dependent: reactive species may act as initiating factors, downstream products of tissue injury, adaptive signals, or amplifiers of inflammatory and metabolic pathways [10].

This narrative review provides a critical synthesis of the evidence linking redox dysregulation to neonatal pathophysiology. It examines the biochemical mechanisms underlying the generation of reactive species, their interactions with inflammatory pathways, and the evidence for oxidative and nitrosative injury in major neonatal disorders. Where possible, evidence from in vitro studies, animal models, observational clinical studies, and interventional trials is considered separately. Particular emphasis is placed on the strengths and limitations of currently available biomarkers and on the emerging hypothesis that the developing skeleton may represent an additional and potentially underestimated target of perinatal redox imbalance.

### 1.1. Review Methodology

This article was designed as a narrative review with a critical and hypothesis‐generating perspective [11]. The literature was searched using PubMed/MEDLINE and Scopus for studies addressing reactive oxygen and nitrogen species, oxidative/nitrosative stress, neonatal disease, and skeletal development. The research covered the last 20 years. Search terms included combinations of “oxidative stress,” “reactive oxygen species,” “reactive nitrogen species,” “free radicals,” “newborn,” “neonate,” “preterm,” “bronchopulmonary dysplasia,” “hypoxic‐ischemic encephalopathy,” “retinopathy of prematurity,” “necrotizing enterocolitis,” “congenital heart disease,” “hyperbilirubinemia,” “bone,” “osteoblast,” “osteoclast.”

We considered experimental studies, animal models, observational clinical studies, clinical trials, systematic reviews, and major narrative reviews addressing redox biology, neonatal OS, and skeletal development. Evidence was synthesized narratively according to the biological mechanism, organ system, and study design. Preclinical and clinical evidence were considered separately whenever possible. Because the aim was to provide a critical narrative synthesis rather than an exhaustive systematic review, no formal meta‐analysis or quantitative assessment of the risk of bias was performed.

## 2. FRs and Other Reactive Species: Biochemical Sources, Physiological Functions, and Molecular Damage

FRs are highly reactive molecules or ions due to their unpaired outer electrons. Although this electronic configuration often confers chemical reactivity, FRs differ markedly in stability, half‐life, diffusion distance, and biological activity. Moreover, FRs should not be considered synonymous with ROS or RNS because both comprise radical and non‐radical species. Radical ROS include superoxide (O_2_•^−^), hydroxyl (•OH), hydroperoxyl (HO_2_•), alkoxyl (RO•), and peroxyl (ROO•) radicals, whereas hydrogen peroxide (H_2_O_2_), hypochlorous acid (HOCl), singlet oxygen (^1^O_2_), ozone (O_3_), and lipid hydroperoxides are non‐radical ROS. Similarly, nitric oxide (NO•) and nitrogen dioxide (NO_2_•) are radical RNS, whereas peroxynitrite (ONOO^−^), nitrous acid (HNO_2_), and dinitrogen trioxide (N_2_O_3_) are non‐radical RNS (Table 1).

**Table 1:** Major radical and non‐radical reactive species, their principal sources, and relevant cellular or tissue contexts.

Class	Reactive species	Formula	Principal biological sources	Relevant cellular or tissue contexts
ROS	Superoxide	O_2_•^−^	Mitochondrial electron transport; NADPH oxidases; xanthine oxidoreductase	Phagocytes, endothelium, epithelium, neurons, muscle, and bone‐lineage cells
ROS	Hydroperoxyl radical	HO_2_•	Protonation of superoxide; lipid environments	Cellular membranes and acidic intracellular compartments
ROS	Hydroxyl radical	•OH	Metal‐catalyzed reactions involving H_2_O_2_	Generated locally near DNA, proteins, lipids, or metal‐binding sites
ROS	Peroxyl radical	ROO•	Propagation of lipid and organic‐substrate oxidation	Lipid membranes and lipoproteins
ROS	Alkoxyl radical	RO•	Decomposition or reduction of organic peroxides	Lipid membranes and sites of organic‐peroxide metabolism
ROS	Hydrogen peroxide	H_2_O_2_	Dismutation of superoxide; oxidases; peroxisomes	Multiple cell types and redox‐signaling compartments
ROS	Singlet oxygen	^1^O_2_	Photochemical reactions; enzymatic and inflammatory processes	Light‐exposed tissues and activated inflammatory environments
ROS	Hypochlorous acid	HOCl	Myeloperoxidase‐dependent oxidation of chloride	Neutrophils and inflamed tissues
ROS	Ozone	O_3_	Primarily environmental; possibly selected inflammatory reactions	Predominantly respiratory epithelium after environmental exposure
RNS	Nitric oxide	NO•	nNOS, eNOS, and iNOS	Endothelium, nervous system, immune cells, muscle, and bone cells
RNS	Nitrogen dioxide	NO_2_•	Secondary reactions involving nitrite, peroxynitrite, or inflammatory oxidants	Inflamed tissues and lipid compartments
RNS	Peroxynitrite	ONOO^−^	Reaction of NO• with O_2_•^−^	Mitochondria, endothelium, nervous tissue, and inflammatory sites
RNS	Dinitrogen trioxide	N_2_O_3_	Reactions involving NO• and nitrogen dioxide	Sites of nitrosation and NO•‐dependent signaling
RNS	Nitrous acid	HNO_2_	Protonation of nitrite in acidic environments	Acidic compartments and inflamed or ischemic tissues

*Note*: The tissue contexts indicate sites in which production or biological effects have been investigated and should not be interpreted as tissue‐specific distributions. Because several reactive species have extremely short half‐lives, their presence is frequently inferred from secondary reaction products or molecular modifications rather than measured directly.

Abbreviations: RNS, reactive nitrogen species; ROS, reactive oxygen species.

Reactive species are continuously generated under physiological conditions. Mitochondria produce superoxide at several sites within the electron transport system, particularly during changes in substrate availability, membrane potential, oxygen tension, or metabolic demand. The rate and biological relevance of mitochondrial ROS production are therefore variable and depend on the cell type and metabolic state rather than representing a fixed proportion of oxygen consumption [12]. Superoxide can undergo spontaneous or superoxide dismutase–catalyzed dismutation to H_2_O_2_ [9, 13]. In contrast to the highly reactive and diffusion‐limited hydroxyl radical, H_2_O_2_ is relatively stable and can participate in regulated redox signaling. H_2_O_2_, in turn, can be converted to the hydroxyl radical (•OH), which is the most reactive ROS and can indiscriminately damage almost all types of biomolecules [14, 15].

NADPH oxidases constitute another major source of ROS. In phagocytes, NOX2‐dependent production of superoxide supports antimicrobial defense during the respiratory burst. Other NOX isoforms are expressed in endothelial, epithelial, neuronal, muscular, and bone‐lineage cells, where controlled ROS production participates in oxygen sensing, vascular regulation, proliferation, differentiation, migration, and extracellular matrix remodeling [9, 16, 17]. Additional physiological sources include xanthine oxidoreductase, cytochrome P450 enzymes, cyclooxygenases and lipoxygenases, peroxisomal metabolism, and oxidative protein folding in the endoplasmic reticulum. Reactive species may also arise from interactions with exogenous agents, including ionizing and ultraviolet radiation, air pollutants, tobacco smoke, xenobiotics, and selected pharmacological treatments [18].

RNS are generated principally through nitric oxide synthases [19, 20]. Neuronal NOS (nNOS/NOS1) and endothelial NOS (eNOS/NOS3) are generally constitutively expressed and produce NO• in a calcium‐dependent manner, whereas inducible NOS (iNOS/NOS2) can generate larger and more sustained amounts of NO• following inflammatory stimulation [21]. At physiological concentrations, NO• regulates vascular tone, neurotransmission, platelet activity, cellular signaling, and host defense. When NO• and superoxide are generated simultaneously within the same biological compartment, they can react to form peroxynitrite. Peroxynitrite and its secondary products can oxidize lipids and nucleic acids, impair mitochondrial and enzymatic functions, and nitrate protein tyrosine residues [22, 23].

Exogenous stimuli also trigger the generation of FR. For example, during the neonatal phase, therapies such as supplemental oxygen can intensify FR‐mediated injury [6, 24, 25]. The genesis of OS changes with the duration of exposure: acute hyperoxia increases mitochondrial ROS, while chronic hyperoxia impairs NO signaling by reducing the cGMP level [26].

The biological effects of reactive species do not follow a single linear progression from generation to cellular damage. Their actions depend on the producing and target cell, the enzymatic source, the chemical identity and concentration of the species, its subcellular localization, the duration of exposure, and the availability of antioxidant and repair systems.

FRs cause cellular damage in several ways, reacting with all biological molecules, including lipids, proteins, and nucleic acids [14]. One of the main mechanisms is the lipid peroxidation of polyunsaturated fatty acids in cell membranes [27]. Peroxidation begins when a FR attacks and abstracts a hydrogen atom from a fatty acid, producing a lipid peroxyl radical. Unless terminated by antioxidant mechanisms, this reaction can propagate through adjacent lipids, alter membrane properties, and generate secondary electrophilic products, including malondialdehyde (MDA) and 4‐hydroxynonenal [10, 28]. The F2‐isoprostanes are prostaglandin‐like products, which originate from in vivo and in vitro peroxidation of arachidonic acid and phospholipids. These prostanoids are less reactive and unstable than other peroxidation products such as aldehydes or peroxyl radicals, so they can be measured using methods such as the gas chromatography coupled to mass spectrometry (MS) technique, liquid chromatography coupled to MS (LC‐MS), and immunological assays [29].

Protein oxidation comprises several distinct processes rather than a single pathway. Reactive species may induce oxidation of amino‐acid side chains, protein carbonyl formation, thiol oxidation, nitration, fragmentation, cross‐linking, altered enzymatic activity, and changes in protein folding or degradation. Some modifications, particularly reversible cysteine oxidation, participate in physiological signaling, whereas irreversible or excessive modifications may impair cellular function [30].

Oxidative nucleic acid damage results from the abstraction of hydrogen atoms from the deoxyribose sugar backbone, resulting in strand breaks or additions to the double bonds of DNA bases [9]. Guanine is particularly susceptible to oxidation, generating products such as 8‐oxo‐7,8‐dihydroguanine and its nucleoside derivative, 8‐oxo‐2^′^‐deoxyguanosine. Both nuclear and mitochondrial DNA may be affected, although susceptibility depends on local reactive species production, chromatin or nucleoid organization, antioxidant protection, and DNA repair capacity [31]. This event causes base changes that can lead to mutations that contribute, among other, to carcinogenesis and other genetic diseases [14, 32]. Indeed, as early as 2005, Spector et al. [33], analyzing 54,795 children, demonstrated a significantly higher rate of childhood cancer among children who received O_2_ for 3 min or more at birth [33].

Biomarkers used to investigate these processes should be classified according to what they measure. F_2_‐isoprostanes, lipid hydroperoxides, MDA, and 4‐hydroxynonenal reflect different aspects of lipid oxidation; protein carbonyls and advanced oxidation protein products indicate protein oxidation; and oxidized guanine derivatives are used as indices of nucleic‐acid oxidation. Protein‐bound 3‐nitrotyrosine is commonly used as a marker of nitrating reactions, whereas nitrite and nitrate primarily reflect NO• metabolism and should not be regarded as direct markers of molecular damage. The glutathione redox couple, antioxidant enzyme activities, and total antioxidant capacity provide information about redox or antioxidant status but do not directly quantify oxidative injury [34].

No individual biomarker provides a comprehensive or organ‐specific measurement of oxidative or nitrosative stress. Biomarker concentrations may be influenced by the biological matrix, sample collection and storage, analytical method, tissue turnover, repair mechanisms, metabolism, and renal elimination. These limitations are particularly relevant in neonatal studies, in which gestational age, postnatal age, nutritional status, inflammation, oxygen exposure, and clinical interventions may act as important confounding factors.

## 3. The Interplay OS and Inflammation

The relationship between OS and inflammation is not simply sequential but synergistic. Activated inflammatory cells contribute to microbial killing by producing proinflammatory cytokines and ROS, particularly extracellular superoxide, which is then converted to hydrogen peroxide [35]. Pro‐inflammatory signals can also increase mitochondrial ROS production and induce iNOS expression, resulting in sustained NO• generation. When superoxide and NO• are produced within the same compartment, peroxynitrite formation may promote protein nitration, lipid oxidation, mitochondrial dysfunction, and nucleic acid damage [36]. On the other hand, excessive ROS production leads to damage to cellular components and endothelial dysfunction, promoting inflammatory pathways and the transition from acute to chronic inflammation [37]. Persistent ROS production in macrophages influences the polarization towards a pro‐inflammatory phenotype, while in dendritic cells, they positively regulate co‐stimulatory molecules for T cell activation [36, 37]. Finally, ROS levels also influence the differentiation of T cells into pro‐inflammatory ones at the expense of regulatory T cells, which are suppressed [38].

Persistent or dysregulated interactions between reactive species production and inflammatory signaling may create self‐sustaining cycles of cellular injury and immune activation.

## 4. Neonatal Diseases Associated With Redox Dysregulation

The transition from intrauterine to extrauterine life is accompanied by an abrupt increase in oxygen availability, activation of mitochondrial aerobic metabolism, and substantial changes in the cardiovascular, respiratory, and metabolic systems. This physiological transition generates a transient oxidative challenge but should not, by itself, be equated with pathological OS. Redox dysregulation arises when the production of reactive oxygen and nitrogen species exceeds or alters the spatial and temporal control provided by antioxidant, redox‐regulatory, and molecular repair systems. The risk of redox dysregulation is particularly high in preterm infants. Preterm birth interrupts the late‐gestational accretion and placental transfer of several antioxidant and nutritional factors and occurs before the complete maturation of endogenous antioxidant systems. The expression and activity of superoxide dismutases, catalase, glutathione peroxidases, and glutathione‐dependent pathways vary according to gestational age, tissue, and developmental stage; therefore, antioxidant immaturity should not be regarded as a uniform deficiency affecting all organs to the same extent [39, 40]. Oxygen exposure represents an important but context‐dependent contributor. Its biological effects depend on the inspired oxygen concentration, duration of exposure, developmental stage, target organ, and concomitant inflammatory or ischemic injury. In the immature retina and lung, excessive oxygen exposure may disrupt hypoxia‐inducible factors and vascular endothelial growth factor signaling, contributing to vascular growth arrest and subsequent pathological responses. Approximately 5%–10% of newborns require supplemental oxygen in the delivery room; guidelines suggest at least minimizing oxygen administration to reduce related complications [41].

The fetal redox environment may also be modified before birth. Maternal diabetes, placental insufficiency and fetal growth restriction, twin‐to‐twin transfusion syndrome, maternal inflammation, and other pregnancy complications have been associated with altered oxidative damage biomarkers, antioxidant capacity, or redox‐regulated placental pathways. However, the available evidence is heterogeneous and is derived largely from placental, cord blood, or neonatal biomarker studies [6, 42–45].

### 4.1. Lung Diseases

The immature lung is particularly susceptible to redox dysregulation because antioxidants, redox‐regulatory, and repair systems are developmentally regulated, whereas supplemental oxygen, mechanical ventilation, inflammation, infection, and nutritional oxidants may increase the oxidative burden. OS should therefore be considered one component of a multifactorial network contributing to neonatal lung injury rather than an isolated or universally initiating mechanism [46].

Respiratory distress syndrome (RDS) primarily results from structural and biochemical lung immaturity and insufficient surfactant availability due to the developmental immaturity of alveolar type II epithelial cells. It should not be characterized by the absence of type‐II pneumocytes. Surfactant deficiency promotes alveolar instability, atelectasis, impaired gas exchange, and the need for respiratory support [47]. The resulting exposure to oxygen, cyclic alveolar recruitment and derecruitment, mechanical stretch, and inflammatory stimuli may increase ROS and RNS production [48]. Activated neutrophils and macrophages can contribute additional oxidants, proteases, and cytokines.

Bronchopulmonary dysplasia (BPD) does not invariably represent a linear progression from RDS. Contemporary BPD may also occur in extremely preterm infants with initially limited respiratory disease. Its pathogenesis reflects interactions among arrested lung development, oxygen toxicity, mechanical injury, inflammation, infection, impaired growth, and genetic or epigenetic susceptibility [49–51]. Experimental hyperoxia and mechanical ventilation models provide mechanistic evidence that excessive or dysregulated ROS production can impair alveolar epithelial and endothelial function, disrupt growth‐factor signaling, alter extracellular matrix remodeling, and interfere with angiogenesis and alveolarization [52, 53].

In contrast to the fibrosis‐dominant pattern of “old” BPD, contemporary or “new” BPD is characterized predominantly by simplified and enlarged distal airspaces, reduced alveolar septation, and abnormal pulmonary microvascular development, with variable interstitial remodeling and fibrosis [54]. Redox‐sensitive pathways may interact with inflammatory signaling and with hypoxia‐inducible factor and vascular endothelial growth factor pathways, thereby linking oxygen exposure to impaired vascular and alveolar development. Reactive species may also reduce NO bioavailability through superoxide‐dependent NO consumption, peroxynitrite formation, endothelial nitric oxide synthase dysfunction, and disruption of soluble guanylate cyclase–cyclic GMP signaling. These mechanisms may contribute to increased pulmonary vascular resistance and pulmonary hypertension associated with BPD. BPD‐associated pulmonary hypertension should be distinguished from persistent pulmonary hypertension of the newborn, which is a separate clinical and pathophysiological entity affecting predominantly term and late preterm infants [55].

Clinical evidence linking oxidative injury to BPD is derived predominantly from observational studies measuring biomarkers in urine, blood, exhaled breath condensate, or tracheal aspirates. In a prospective study of 60 infants born at less than 30 weeks of gestation or weighing less than 1250 g, urinary 8‐hydroxydeoxyguanosine (8‐OHdG) on day 3 correlated with the duration of mechanical ventilation, while day‐7 concentrations were independently associated with subsequent moderate or severe BPD [56]. In the same study, urinary leukotriene E_4_ (LTE_4_) showed a different temporal pattern. Importantly, 8‐OHdG is a marker of oxidative nucleic acid modification, whereas LTE_4_ is primarily an inflammatory lipid mediator; they should not be presented as equivalent oxidative damage markers.

Products of lipid peroxidation, including lipid hydroperoxides, F_2_‐isoprostanes, and isofurans, have also been reported at higher concentrations in preterm infants who subsequently developed BPD than in infants without BPD [57, 58]. Moreover, lipid peroxide levels have been found associated with respiratory support parameters, including the duration of mechanical ventilation, supporting the role of volu/barotrauma in triggering the inflammatory cascade and amplifying lung injury [57]. A link between the interleukin pathway and OS biomarkers has also been reported. Specifically, IL‐6 and 8‐OHdG levels in serum and tracheal aspirates were found to be higher in VLBW infants with BPD than in those without BPD on the first day after birth [59]. IL‐6 reflects inflammatory signaling, whereas 8‐OHdG reflects oxidative nucleic acid modification.

Taken together, these clinical studies support an association among early inflammation, oxidative molecular modifications, respiratory disease severity, and subsequent BPD. Their interpretation is limited by generally small sample sizes, heterogeneous BPD definitions, differences in biological matrices and sampling times, potential effects of gestational age and renal function, and confounding by oxygen exposure, ventilation, infection, nutrition, and severity of illness. Biomarker studies therefore complement but do not prove the causal mechanisms suggested by experimental models.

Finally, although it is not among the classic free‐radical‐induced neonatal diseases, meconium aspiration syndrome must also be considered. Its pathophysiology is multifactorial and includes airway obstruction, surfactant inactivation, chemical pneumonitis, ventilation–perfusion mismatch, pulmonary vasoconstriction, and, in severe cases, persistent pulmonary hypertension of the newborn [60]. Meconium contains bile acids, bilirubin, cholesterol, free fatty acids, digestive enzymes, cellular debris, and biologically active inflammatory components. The traditional description of meconium as uniformly sterile should be avoided because microbial findings vary according to the clinical and sampling context.

Meconium exposure can recruit neutrophils, stimulate inflammatory mediators, increase oxidative products, impair surfactant function, and damage pulmonary epithelial cells [61, 62]. Animal models also suggest that hypoxemia, inflammation, and reoxygenation may promote oxidative injury beyond the lung [63, 64]. Clinical studies have reported an altered oxidant–antioxidant balance in newborns exposed to meconium‐stained amniotic fluid, but these findings are limited and do not establish OS as the primary mechanism of MAS [65, 66]. Accordingly, redox dysregulation should be presented as a potential contributor that interacts with mechanical obstruction, inflammation, hypoxemia, and pulmonary vascular dysfunction rather than as an independently demonstrated cause.

#### 4.1.1. Therapeutic Implications

Current prevention and treatment strategies for neonatal lung disease are not designed primarily as free‐radical‐scavenging interventions. Nevertheless, several conventional approaches may indirectly reduce the oxidative burden by limiting upstream injury. These include careful titration of supplemental oxygen, pulse‐oximetry‐guided saturation targets, preferential use of non‐invasive respiratory support when appropriate, lung‐protective ventilation, avoidance of prolonged invasive ventilation, early surfactant treatment in selected infants, infection control, and adequate nutrition [67]. Caffeine may reduce the BPD risk partly by facilitating earlier discontinuation of positive‐pressure ventilation, but a direct antioxidant mechanism has not been established. Similarly, vitamin A supports epithelial integrity and lung development but should not be described simply as a radical scavenger.

Direct antioxidant approaches have produced less consistent clinical results than those suggested by preclinical studies. In a multicenter randomized trial, 391 infants weighing 500–999 g who required mechanical ventilation or nasal continuous positive airway pressure were randomized before 36 h of age to intravenous N‐acetylcysteine or placebo for 6 days. N‐acetylcysteine did not reduce death or BPD, BPD among survivors, oxygen requirement at 28 days, or BPD severity [68]. Trials of recombinant human superoxide dismutase have not provided sufficient evidence of a reduction in BPD or death, although some follow‐up findings have suggested possible effects on later respiratory morbidity [69]. Other candidate interventions with antioxidant or redox‐modulating properties have shown favorable effects in experimental hyperoxia models, but the evidence supporting routine clinical use remains insufficient.

These findings illustrate why successful attenuation of oxidative injury cannot be assumed from antioxidant activity alone. The efficacy of an intervention may depend on its intracellular and tissue distribution, timing relative to the oxidative insult, specificity for the relevant reactive species, and ability to preserve physiological redox signaling. Broad suppression of ROS or RNS could theoretically interfere with host defense, vascular adaptation, angiogenesis, and cellular differentiation. At present, reduction of avoidable oxygen and ventilation‐associated injury remains more firmly supported than routine administration of a direct antioxidant for BPD prevention.

The management of MAS remains primarily supportive and is directed at adequate oxygenation and ventilation, surfactant dysfunction, pulmonary air leak, and associated pulmonary hypertension rather than at direct antioxidant treatment. Redox dysregulation should therefore be presented as a potential secondary contributor interacting with airway obstruction, inflammation, hypoxemia, reoxygenation, and pulmonary vascular dysfunction rather than as an independently demonstrated cause or currently validated therapeutic target.

### 4.2. Brain Injury

The brain is at high risk for FRs injury due to the high amount of polyunsaturated fatty acids and the concomitant lack of antioxidant defenses [70]. However, susceptibility is not uniform across the brain and varies according to gestational age, cell lineage, maturational stage, anatomical region, and the nature and duration of the insult.

White matter injury (WMI) is one of the principal forms of brain injury in very preterm infants and is associated with adverse motor, cognitive, and behavioral outcomes [71]. Its contemporary phenotype is more frequently diffuse and non‐cystic than the classic cystic periventricular leukomalacia described in earlier cohorts. Intraventricular hemorrhage, cerebellar injury, and impaired cerebral growth constitute additional and partly overlapping patterns of preterm brain injury and should be considered separately. Before 32 weeks of gestation, ~90% of oligodendrocytes are in the pre‐oligodendrocyte stage, cells that are extremely sensitive to free‐radical damage caused by ischemia [72]. Current evidence supports a multifactorial model in which vascular immaturity and impaired perfusion interact with maturation‐dependent cellular vulnerability. In cellular and animal models, activated microglia and infiltrating macrophages can generate superoxide through NADPH oxidases, produce NO through an inducible nitric oxide synthase, and release inflammatory cytokines. The interaction between superoxide and NO may generate peroxynitrite, contributing to protein nitration, mitochondrial impairment, and pre‐oligodendrocyte injury [73].

Clinical evidence is derived primarily from observational biomarker studies. In a study of very‐low‐birth‐weight infants with WMI assessed by magnetic resonance imaging at term equivalent age, lipid peroxidation products were increased in cerebrospinal fluid, with higher concentrations reported in infants with more severe cystic periventricular leukomalacia and ventriculomegaly than in those with less severe white matter abnormalities [74].

Ahola et al. [68] measured plasma 8‐isoprostane on postnatal days 3 and 7 in 83 extremely‐low‐birth‐weight infants enrolled in a randomized trial of N‐acetylcysteine. The concentrations increased more markedly in infants who subsequently developed periventricular leukomalacia. Ascorbyl radical concentrations, measured by electron‐spin resonance spectroscopy in 61 infants, were also higher on day 3 among infants who later developed periventricular leukomalacia, but the between‐group pattern was not sustained [68]. These findings preceded the neuroimaging diagnosis but were not specific to cerebral injury because plasma 8‐isoprostane was also associated with BPDor death.

Matthews et al. [75] measured plasma F_2_‐isoprostanes on postnatal days 14 and 28 in 136 infants born at or before 28 weeks’ gestation. Increasing concentrations during the first postnatal month were associated with greater respiratory morbidity at term‐equivalent age and lower cognitive, communication, and motor scores at 12 months’ corrected age [75].

In a prospective single‐center study of 44 infants born before 28 weeks’ gestation, Coviello et al. [76] showed that peripheral plasma isoprostanes measured within 48 h after birth were positively associated with WMIs at the term of an equivalent age. In a separate prospective cohort of extremely preterm infants, plasma isoprostanes and non‐protein‐bound iron were evaluated for early cerebral activity recorded by amplitude‐integrated electroencephalography. Higher isoprostane concentrations were associated with less mature or depressed background activity [77].

Collectively, these studies indicate that lipid peroxidation products in cerebrospinal fluid or plasma are associated with structural brain abnormalities, altered early cerebral activity, or later developmental outcomes in selected preterm populations. However, circulating isoprostanes are not brain‐specific, may originate from other injured organs, and may reflect the overall illness severity or oxygen exposure. Small sample sizes, heterogeneous neuroimaging definitions, different sampling times, and residual confounding currently preclude their use as validated individual predictors of WMI, intraventricular hemorrhage, or neurodevelopmental impairment.

In term and near‐term infants, hypoxic–ischemic encephalopathy (HIE) may follow an acute or prolonged reduction in cerebral oxygen delivery and blood flow. The evolution of injury is more accurately described as primary energy failure, a latent phase, secondary energy failure, and, in some infants, a tertiary phase rather than as three discrete stages of oxygen deprivation, energy depletion, and reoxygenation [78]. During the hypoxic–ischemic insult, impaired oxidative phosphorylation reduces adenosine triphosphate production, causing the failure of ion pumps, membrane depolarization, cytotoxic edema, excessive glutamate release, calcium influx, and, when severe, necrotic cell death. Mitochondrial electron transport dysfunction and xanthine oxidase activity may promote superoxide and H_2_O_2_ generation during hypoxia and particularly during reoxygenation [79]. Neuronal nitric oxide synthase activation can increase NO production early after excitotoxic calcium influx, whereas inducible nitric oxide synthase is more commonly associated with a later inflammatory response. NO may have physiological vascular effects, but its reaction with superoxide generates peroxynitrite, which can promote protein nitration, lipid oxidation, nucleic acid damage, and mitochondrial dysfunction.

The latent phase, which generally follows initial resuscitation, is characterized by partial recovery of oxidative metabolism and provides a time‐limited therapeutic window. Secondary energy failure develops over the subsequent hours and is associated with mitochondrial dysfunction, oxidative and nitrosative injury, excitotoxicity, seizures, inflammation, and apoptotic or regulated necrotic cell death. A later tertiary phase may involve persistent inflammation, impaired repair, epigenetic alterations, and disturbed neurogenesis and connectivity. The relative contribution of each pathway varies with the severity, duration, and timing of the insult and with the affected brain region [80].

Much of the evidence identifying superoxide, H_2_O_2_, hydroxyl radicals, NO, and peroxynitrite in HIE derives from cell culture and neonatal animal models using redox‐sensitive probes, spin‐trapping techniques, molecular oxidation products, or protein nitration. Direct measurement in the human neonatal brain remains difficult because these species are short‐lived. Clinical studies generally rely on circulating, urinary, cerebrospinal fluid, or magnetic resonance spectroscopy biomarkers, which provide indirect evidence of cerebral redox injury [81].

#### 4.2.1. Therapeutic Implications

Therapeutic hypothermia initiated within 6 h after birth remains the established treatment for eligible term and near‐term infants with moderate or severe HIE. Hypothermia reduces cerebral metabolic demand and modifies multiple downstream pathways, including excitotoxicity, mitochondrial dysfunction, inflammation, and oxidative injury, but it is not a selective antioxidant treatment. It provides incomplete protection, particularly after severe or prolonged insults, and is not currently established as a routine therapy for preterm WMI.

Several adjunctive redox‐modulating strategies have shown neuroprotective effects in experimental models, including allopurinol, melatonin, N‐acetylcysteine, erythropoietin, noble gases, and mitochondria‐targeted agents. Translation to clinical benefit remains uncertain. Despite encouraging preclinical and early‐phase findings, two phase III randomized controlled trials—the HEAL and PAEAN trials—found that high‐dose erythropoietin administered as an adjunct to therapeutic hypothermia did not reduce death or moderate‐to‐severe neurodevelopmental disability at approximately 2 years in neonates with moderate‐to‐severe HIE. These findings do not support the routine clinical use of adjunctive erythropoietin in this setting [82, 83]. The phase III ALBINO trial is evaluating early allopurinol in addition to standard care; definitive primary outcome results were not yet available at the time of this revision [84]. Evidence for melatonin and other direct antioxidant interventions remains insufficient for routine neonatal neuroprotection.

As in the lung, complete suppression of reactive species is neither feasible nor necessarily desirable. NO contributes to cerebral vascular regulation, while controlled ROS production participates in cellular signaling, immune responses, and repair. Neuroprotective strategies should therefore aim to limit pathological compartment‐specific redox dysregulation within an appropriate therapeutic window while preserving physiological signaling.

### 4.3. Retinopathy of Prematurity (ROP)

The retina can be considered an extension of the brain and, like the brain, is an organ that consumes a lot of oxygen and is rich in polyunsaturated fatty acids, which are exposed to lipid peroxidation. During normal development, controlled ROS and RNS production support retinal vascularization, oxygen sensing, cellular differentiation, and angiogenic signaling. Reactive species generation in the developing retina is therefore not intrinsically pathological; its effects depend on concentration, cellular source, subcellular localization, developmental stage, and oxygen context [85–90]. ROP is a multifactorial neurovascular disorder of the incompletely vascularized retina. Lower gestational age and birth weight are the major determinants of susceptibility, while oxygen exposure and fluctuations, impaired postnatal growth, inflammation, infection, blood transfusions, and nutritional factors may modify the risk [85]. ROP is conventionally described as a biphasic process. In phase 1, premature exposure to the extrauterine oxygen environment, potentially compounded by supplemental oxygen and oxygen fluctuations, suppresses oxygen‐regulated vascular survival signals and interrupts physiological retinal vascular development. In contemporary ROP, this process is characterized mainly by delayed peripheral vascularization, although vaso‐obliteration can occur under more severe hyperoxic conditions. As the neural retina continues to mature and its metabolic requirements increase, the avascular retina becomes relatively hypoxic. During phase 2, stabilization of hypoxia‐inducible factors and increased expression of VEGF and other angiogenic mediators promote pathological neovascularization at the junction between the vascularized and avascular retina [85, 88]. Evidence implicating redox‐sensitive signaling in ROP derives predominantly from cultured retinal cells and animal models of oxygen‐induced retinopathy. These models include constant hyperoxia in neonatal mice and fluctuating oxygen exposure in neonatal rats. Constant exposure to 75% oxygen in the mouse model produces central vaso‐obliteration, followed by relative retinal hypoxia and intravitreal neovascularization upon discontinuation of oxygen supplementation and exposure to room air. In contrast, fluctuating‐oxygen rat models more closely reproduce delayed physiological retinal vascular development and peripheral avascular retina observed in contemporary human ROP [88]. Neither model fully reproduces the oxygen exposure, developmental timing, or clinical heterogeneity of ROP in preterm infants. In these experimental models, retinal endothelial cells, Müller cells, astrocytes, microglia, and neurons contribute differently to redox‐regulated vascular responses. Mitochondrial ROS, NADPH oxidases, endothelial nitric oxide synthase, NO, and peroxynitrite have been implicated in apoptosis, delayed retinal vascularization, vaso‐obliteration, and pathological neovascularization [87–89]. Wang et al. [88] summarized experimental evidence that redox‐sensitive pathways—including HIF‐1α, VEGF, JAK/STAT3, phospholipase A_2_, PPARγ, and erythropoietin signaling—may have beneficial or pathological effects depending on the disease phase and retinal cell involved.

Direct measurement of short‐lived reactive species in the retina of living preterm infants is not feasible. Human evidence therefore relies on systemic biomarkers, ocular samples obtained in exceptional circumstances, and associations between clinical exposures and ROP.

Saugstad and Rognum [90] measured hypoxanthine in post‐mortem vitreous humor from premature infants who died with RDS. Hypoxanthine provides a substrate for xanthine oxidoreductase‐dependent generation of superoxide and H_2_O_2_ during reoxygenation. Elevated levels of total hydroperoxides, advanced oxidative protein products, and nonprotein‐bound iron have been found in cord blood of babies at risk for FR diseases and consequently of ROP [91]. Banjac et al. [86] performed a prospective cohort study of 59 infants born at 33 weeks’ gestation or earlier. Total oxidant status (TOS), MDA, total antioxidant status (TAS), and paraoxonase‐1 activity were measured spectrophotometrically. TOS and MDA were higher in the treatment‐requiring ROP group, whereas TAS and paraoxonase‐1 activity did not differ significantly between groups [86]. Furthermore, it has been shown that the retinal antioxidant enzyme content is low in cases of ROP [87].

Overall, clinical evidence supports an association between systemic redox imbalance and ROP severity in selected populations but remains insufficient to identify a specific circulating biomarker for individual prediction. Systemic markers cannot establish retinal origin, and their concentrations are influenced by gestational age, respiratory disease, oxygen exposure, ventilation, infection, nutrition, transfusions, and other prematurity‐related complications. No oxidative or nitrosative biomarker currently replaces serial retinal examination or established clinical risk assessment.

#### 4.3.1. Therapeutic Implications

The prevention of ROP primarily targets modifiable upstream exposures. Careful titration of supplemental oxygen, avoidance of sustained hyperoxia and marked oxygen fluctuations, pulse oximetry‐guided saturation targets, adequate nutrition and postnatal growth, infection prevention, and judicious transfusion practices may indirectly reduce oxidative and inflammatory injury. Oxygen restriction must not compromise survival or neurodevelopment; therefore, prevention requires balanced oxygen targeting rather than indiscriminate oxygen minimization.

Laser photocoagulation and intravitreal anti‐VEGF agents treat the angiogenic consequences of phase‐2 ROP but are not direct antioxidant therapies. Antioxidant supplementation has not demonstrated a sufficiently consistent efficacy and safety profile for routine ROP prevention. Historical vitamin E studies suggested a possible reduction in severe ROP but raised concerns regarding sepsis and necrotizing enterocolitis (NEC) [88]. Other interventions, including recombinant superoxide dismutase, N‐acetylcysteine, lutein, zeaxanthin, omega‐3 fatty acids, and melatonin, remain investigational or have yielded inconsistent clinical findings.

Experimental evidence also indicates that broad inhibition of ROS may have different or even opposing effects in the two phases of ROP. Reactive species participate in physiological angiogenesis, oxygen sensing, immune defense, and cell signaling. Redox‐targeted interventions should therefore be phase‐specific and pathway‐specific and should preserve physiological retinal vascularization while limiting pathological neovascularization.

### 4.4. NEC

NEC is a multifactorial inflammatory disease occurring predominantly in preterm infants and most commonly involving the terminal ileum and proximal colon. Prematurity‐related intestinal immaturity, formula feeding, microbial dysbiosis, exaggerated innate immune signaling, impaired microvascular regulation, and episodes of hypoxia–reoxygenation may interact in its pathogenesis [92]. Reactive oxygen and nitrogen species should be regarded as context‐dependent mediators within this network rather than as independently demonstrated initiating causes of NEC [93]. At physiological concentrations, reactive species contribute to intestinal epithelial signaling, mucosal renewal, vascular tone regulation, and antimicrobial defense. In particular, nitric oxide generated constitutively by endothelial nitric oxide synthase supports intestinal perfusion and epithelial‐barrier homeostasis. During excessive or prolonged inflammation, however, inducible nitric oxide synthase may be upregulated in enterocytes and activated immune cells, resulting in sustained nitric oxide production. The reaction of nitric oxide with superoxide generates peroxynitrite, a potent non‐radical oxidant capable of inducing protein tyrosine nitration, lipid oxidation, mitochondrial dysfunction, and DNA damage. Thus, the biological effects of nitric oxide depend on its cellular source, concentration, duration of production, and local availability of superoxide [94]. Most mechanistic evidence supporting these pathways derives from cellular and animal models. In rat intestinal epithelial IEC‐6 cells, exposure to 50 μM peroxynitrite activated the MKK3/6–p38–ATF2 pathway, reduced prosurvival AKT signaling, and promoted enterocyte apoptosis; pharmacological inhibition or silencing of p38 attenuated this response [95]. Experimental NEC models have additionally associated increased inducible nitric oxide synthase activity and nitrotyrosine formation with epithelial apoptosis, impaired mitochondrial respiration, disruption of tight junction proteins, and increased intestinal permeability [94, 95]. These models provide biological plausibility but reproduce only selected components of the heterogeneous human disease. The intestinal epithelial cells of premature infants are extremely sensitive to FRs produced in response to stressful events, such as hypoxia, reperfusion injury, and bacterial colonization. Indeed, inflammatory stimulus activates iNOS, increasing NO production and subsequent peroxynitrite formation [96]. Peroxynitrite, in turn, causes nitrosylation of mitochondrial proteins, inhibiting mitochondrial respiration and activating apoptosis. Finally, NO also causes nitrosylation of barrier lipids and proteins altering the intestinal barrier, leading to a weakening of the barrier function and a decrease in Paneth cells, allowing for increased invasion of harmful bacteria [97]. Subsequently, inflammatory mediators are released, which initiate the inflammatory cascade with the recruitment of PMN to the intestinal wall and further production of ROS [92, 93]. Clinical evidence remains more limited and is based mainly on systemic biomarkers rather than measurements obtained directly from the affected intestinal tissue. In a multicentre prospective cohort of preterm infants born at 24–33 weeks of gestation, non‐protein‐bound iron, total hydroperoxides, and advanced oxidation protein products were measured in cord blood. Mean cord blood concentrations of all three biomarkers were significantly higher in preterm newborns who later developed NEC, demonstrating a strong association between intrauterine OS events and the risk of developing NEC [98].

Current NEC‐prevention strategies—including human milk feeding, standardized feeding protocols, careful antibiotic stewardship, and, in selected settings, probiotic supplementation—may indirectly reduce inflammatory and oxidative burden, but they are not primarily antioxidant treatments [97, 99]. Once NEC is established, bowel rest, gastric decompression, antimicrobial therapy, cardiorespiratory support, and surgery when indicated remain the foundations of treatment. No antioxidant or redox‐targeted intervention has yet demonstrated sufficient efficacy and safety for routine clinical use. Agents such as N‐acetylcysteine, melatonin, and modulators of nitric oxide or related signaling pathways have shown potentially protective effects predominantly in experimental models. Translation requires caution because physiological ROS and nitric oxide signaling also support intestinal perfusion, epithelial adaptation, and antimicrobial defense; indiscriminate suppression of reactive species could therefore be ineffective or harmful.

### 4.5. Neonatal Jaundice

Hyperbilirubinemia is one of the most common conditions affecting neonates. In neonatal red blood cells, OS mediated by FRs contributes to eryptosis, a form of programed cell death in erythrocytes that results from alterations in membrane integrity [100]. Neonatal erythrocytes may act both as targets and as sources of oxidative injury. Their susceptibility is influenced by membrane lipid composition, relatively limited antioxidant capacity, hemoglobin oxidation, and, in specific conditions, enzymatic defects such as glucose‐6‐phosphate dehydrogenase deficiency. Superoxide and hydrogen peroxide can be generated during hemoglobin autoxidation, while the release of redox‐active iron may promote hydroxyl‐radical formation through Fenton chemistry. Oxidative membrane damage, intracellular calcium accumulation, phosphatidylserine exposure, cell shrinkage, and membrane blebbing may promote eryptosis, a programed erythrocyte death phenotype [101].

At mildly elevated levels, bilirubin has been described as a physiological antioxidant. Albumin‐bound unconjugated bilirubin can scavenge peroxyl radicals and inhibit lipid peroxidation, thereby contributing to physiological plasma antioxidant capacity [102]. Conversely, marked increases in unbound bilirubin may promote cellular dysfunction, particularly in the nervous system. Its biological effects depend not only on total serum bilirubin but also on albumin binding, bilirubin–albumin molar ratio, gestational and postnatal age, acidosis, sepsis, and the presence of competing albumin‐binding substances. Therefore, total bilirubin concentration alone does not define whether its net effect is antioxidant or cytotoxic. Bulut et al. [103] investigated the impact of hyperbilirubinemia on DNA damage as well as on total oxidant and antioxidant status in neonates undergoing phototherapy. Plasma 8‐hydroxy‐2^′^‐deoxyguanosine, a marker of oxidative DNA modification, and TOS were higher in hyperbilirubinemic infants, particularly when total bilirubin exceeded 16 mg/dL. TAS was also higher and correlated positively with bilirubin concentration, whereas the calculated OS index did not differ significantly between groups [103].

These apparently divergent findings are consistent with the proposed dual antioxidant and potentially damaging associations of bilirubin. Neonatal erythrocytes act both as targets and sources of FRs, particularly through the Fenton and Haber‐Weiss reactions.

Phototherapy introduces an additional source of complexity. By converting bilirubin into more readily excretable photoisomers, it remains the established treatment for clinically significant unconjugated hyperbilirubinemia. At the same time, light exposure may generate photo‐oxidation products and transiently modify circulating OS biomarkers. Consequently, studies evaluating samples obtained after phototherapy cannot readily distinguish the redox effects of hyperbilirubinemia from those of the treatment. This limitation does not challenge the clinical benefit of appropriately indicated phototherapy but emphasizes the need to report sampling in relation to its initiation and duration.

A study of 100 neonates with indirect hyperbilirubinemia compared phototherapy plus oral vitamin E at 25 mg/kg/day for 3 days with phototherapy alone. The vitamin E group had lower total serum bilirubin and higher total antioxidant capacity on the third day as well as a shorter reported duration of phototherapy [104]. Current evidence is insufficient to recommend vitamin E or other antioxidant supplementation specifically for the treatment of neonatal hyperbilirubinemia. Management should remain directed toward identifying the underlying cause and reducing bilirubin exposure through appropriate feeding support, phototherapy, intravenous immunoglobulin in selected immune‐mediated hemolytic conditions, or exchange transfusion when indicated. Future studies of redox‐targeted interventions should stratify infants by gestational age and etiology, measure both total and unbound bilirubin, standardize sampling relative to phototherapy, and assess safety and clinically meaningful neurodevelopmental outcomes.

### 4.6. Congenital Heart Diseases (CHDs)

OS appears to play a role in both the pathogenesis and early detection of CHD during prenatal and early postnatal stages. The relationship between redox biology and CHD differs according to the developmental stage. During embryogenesis, physiological concentrations of ROSparticipate in cardiomyocyte differentiation, growth factor signaling, vascular development, and cardiac morphogenesis. Excessive or spatially dysregulated reactive species production may interfere with these processes, but human evidence that OS independently causes CHD remains limited. After birth, cyanotic CHD creates a different redox context in which chronic or intermittent hypoxemia, altered tissue perfusion, inflammation, and subsequent reoxygenation may promote oxidative and nitrosative molecular modifications. OS should therefore be considered a potential modifier of tissue injury and clinical severity rather than a unifying cause of structurally heterogeneous cardiac malformations.

Escobar‐Diaz et al. [105] conducted a retrospective case–control pilot study including amniotic fluid samples from fetuses with postnatally confirmed isolated major CHD. Amniotic fluid ortho‐tyrosine concentrations were higher in CHD pregnancies than in controls, whereas 3‐nitrotyrosine and the normalized ortho‐tyrosine/phenylalanine ratio did not differ significantly. The highest ortho‐tyrosine concentrations were observed in fetuses predicted to develop extreme postnatal cyanosis, particularly those with transposition physiology [105]. More broadly, OS is implicated in the embryogenesis of CHD, where environmental factors—such as maternal hyperhomocysteinemia linked to inadequate folate/B12 status—may elevate ROS levels and impair the antioxidant glutathione pathway [106]. Hyperhomocysteinemia may plausibly influence endothelial function, methylation reactions, and glutathione metabolism, and OS represents one of several potential mechanistic links. While much research on OS biomarkers in CHD focuses on postoperative settings, general neonatal OS biomarker studies underscore that oxidation of phenylalanine produces byproducts—o‐Tyr, m‐Tyr, 3‐nitro‐tyrosine (3NO_2_‐Tyr), and 3‐chlorotyrosine (3Cl‐Tyr)—which are measurable in amniotic fluid, plasma, urine, and cerebrospinal fluid and serve as reliable indicators of oxidative and nitrosative stress [107].

Ortho‐tyrosine and meta‐tyrosine are generated through hydroxyl‐radical‐mediated oxidation of phenylalanine and primarily indicate protein oxidative modification. In contrast, 3‐nitrotyrosine reflects protein tyrosine nitration associated with reactive nitrogen species, particularly peroxynitrite‐derived pathways, whereas 3‐chlorotyrosine is considered a marker of myeloperoxidase‐derived hypochlorous acid activity. These stable molecular products can be measured in biological matrices such as amniotic fluid, plasma, urine, and cerebrospinal fluid, but their concentrations are matrix‐ and context‐dependent, and they should not be described interchangeably as direct measurements of FRs [107].

A recent prospective case–control study by Kurt et al. [108] investigated OS markers, antioxidant capacity, thiol/disulfide homeostasis, and heme oxygenase‐1 (HO‐1) levels in term neonates with cyanotic CHD compared to healthy controls. The serum TOS and the calculated OS index were higher in the cyanotic CHD group. TAS was also increased, possibly reflecting a compensatory response, whereas native thiol, total thiol, and heme oxygenase‐1 concentrations were lower. These findings suggest an association among cyanotic physiology, systemic redox imbalance, and illness severity.

Receiver operating characteristic analysis showed the diagnostic potential of these biomarkers: TOS (cut‐off > 12.96; sensitivity 96.83%) and OS index (cut‐off > 0.86; specificity 86.96%) were strong predictors, while HO‐1 demonstrated excellent diagnostic accuracy (AUC ≈ 0.980) [108].

However, TOS and TAS are composite assays and do not identify specific reactive species, their cellular sources, or the organs undergoing oxidative injury. Similarly, the circulating HO‐1 concentration does not necessarily reflect its tissue expression or enzymatic activity in cardiomyocytes, vascular endothelial cells, or other organs. Biomarker concentrations may also be influenced by the type of CHD, severity and duration of hypoxemia, acidosis, prostaglandin treatment, oxygen administration, tissue hypoperfusion, infection, nutrition, and timing of blood collection. No circulating redox biomarker is currently validated to guide neonatal CHD treatment, and no antioxidant therapy has demonstrated sufficient clinical benefit to support routine use specifically for cyanotic CHD. Antioxidant supplementation or pharmacological modulation of HO‐1 and thiol pathways remains experimental. Future studies should distinguish prenatal mechanisms from postnatal cyanotic and perioperative injury, characterize lesion‐specific and tissue‐specific pathways, include serial sampling before and after changes in oxygenation, and determine whether redox biomarkers improve neurological, cardiac, or survival outcomes.

### 4.7. Redox Dysregulation and Early Skeletal Development

Bone development is a dynamic process involving mesenchymal cell differentiation, chondrogenesis, endochondral or intramembranous ossification, matrix production, mineralization, and coordinated osteoblast–osteoclast activity. ROSparticipate in the physiological regulation of these processes, whereas excessive or persistent production may damage cellular macromolecules and disrupt skeletal development. Preterm infants are particularly vulnerable to impaired bone mineral accrual because most fetal calcium and phosphorus transfer occur during the third trimester. Nevertheless, metabolic bone disease of prematurity is primarily associated with inadequate mineral and protein intake, prolonged parenteral nutrition, rapid postnatal growth, immobilization, and exposure to corticosteroids or loop diuretics [109, 110]. Redox dysregulation should therefore be considered a potential contributing factor rather than an established primary cause.

Mechanistically, reactive species may affect three interconnected cellular compartments: osteoblast‐lineage cells, osteoclast‐lineage cells, and growth‐plate chondrocytes. In osteoprogenitors, canonical Wnt signaling stabilizes β‐catenin and promotes T‐cell factor/lymphoid enhancer factor‐dependent transcription, RUNX2 activation, osteoblast differentiation, and matrix production. Experimental OS can divert β‐catenin from osteogenic transcription toward forkhead box O‐mediated stress responses, thereby reducing osteoblast differentiation and favoring cellular senescence, apoptosis, or adipogenic commitment [111]. In osteoclast precursors, by contrast, transient ROS production is a physiological component of RANKL–TRAF6–Rac1–NADPH oxidase 1 signaling and supports osteoclast differentiation through mitogen‐activated protein kinase and NFATc1 activation [112]. Excessive or sustained ROS production may amplify osteoclastogenesis and bone resorption. Thus, reactive species may impair bone accrual by simultaneously reducing osteoblast‐mediated formation and increasing osteoclast‐mediated resorption, although these mechanisms have been demonstrated mainly in cellular and animal models.

Longitudinal bone growth additionally depends on the orderly progression of growth‐plate chondrocytes through resting, proliferative, prehypertrophic, and hypertrophic stages. SOX9 regulates chondrocyte differentiation and cartilage matrix production, whereas RUNX2, matrix remodeling, and vascular invasion contribute to hypertrophic differentiation and replacement of cartilage by bone. Niu et al. [113], using chick embryos and cultured chondrocytes, showed that alkoxyl radicals generated during phospholipid peroxidation modified SOX9 and promoted its degradation, impairing chondrogenesis and endochondral bone development. Iron chelation and modulation of transferrin receptor 1 and divalent metal transporter 1 attenuated these effects [113].

Additional models support the developmental sensitivity of bone to redox perturbations. In iron‐loaded mice, Tsay et al. [114] observed reduced bone mass and altered remodeling associated with OS, partially attenuated by antioxidant treatment. In chick embryos, hydrogen peroxide exposure altered antioxidant responses and reduced the expression of genes involved in cartilage and bone formation, including COL1A2, COL2A1, BMP2, BGLAP, and RUNX2, resulting in impaired embryonic growth and skeletal development [115]. Studies in wild‐type and catalase‐overexpressing mice further indicate that hydrogen peroxide affects cortical and trabecular bone turnover in an age‐dependent manner [116, 117]. Nitric oxide also exerts context‐dependent effects: sustained inducible nitric oxide synthase activation in inflammatory models suppresses osteoblast function and promotes apoptosis [118], whereas controlled nitric oxide availability may support osteogenic differentiation of mesenchymal or embryonic stem cells [119].

Taken together, these experimental findings provide a biologically plausible framework in which excessive redox activity may impair osteoblast differentiation, enhance osteoclast activity, and disrupt growth‐plate chondrogenesis. However, most evidence derives from cultured cells, chick embryos, and rodent models, often involving exposures not directly comparable with neonatal clinical conditions.

Human evidence directly connecting perinatal redox status with postnatal skeletal development is currently limited to exploratory observational studies. In a preliminary longitudinal cohort, Perrone et al. [120] demonstrate that perinatal OS, as measured through biochemical markers in umbilical cord blood, is inversely associated with early skeletal development, as assessed by REMS‐derived Z‐scores in the first year of life. Higher levels of oxidative damage markers (8‐hydroxy‐2^′^‐deoxyguanosine and advanced oxidation protein products) were inversely associated with REMS‐derived Z‐scores at 6 and 12 months [120].

Furthermore, a significant correlation was found between total antioxidant capacity and key auxological parameters at birth, including head circumference, suggesting a broader role of the oxidative balance in neonatal growth regulation [120–122].

By integrating biochemical markers of OS in cord blood with longitudinal auxological data and bone quality assessment through REMS, a non‐invasive, radiation‐free technology, it has been provided novel evidence that early oxidative balance influences growth trajectories and bone quality during the first year of life.

No clinical trial has established that antioxidant supplementation prevents metabolic bone disease or improves skeletal development in preterm or term infants. Current preventive strategies should remain focused on adequate calcium, phosphorus, protein, and vitamin D provision; appropriate human milk fortification; biochemical monitoring of high‐risk infants; promotion of safe physical activity; and reduction of avoidable medication‐related risk. These measures may indirectly support redox homeostasis, but their efficacy is not mediated exclusively through antioxidant mechanisms.

Experimental antioxidants, iron chelation, or pharmacological modulation of nitric oxide and redox‐sensitive pathways cannot currently be recommended for neonatal bone protection. Excessive suppression of reactive species could also interfere with physiological signaling required for osteoclast differentiation, angiogenesis, host defense, and normal remodeling. Future studies should combine standardized oxidative damage biomarkers with established biochemical indices of bone metabolism, validated imaging, growth data, fracture outcomes, and longitudinal assessment. Inclusion of preterm infants at different gestational ages will be necessary to determine whether redox dysregulation contributes independently to metabolic bone disease beyond mineral deficiency, illness severity, nutrition, and medication exposure.

### 4.8. Limitations of Oxidative and Nitrosative Stress Biomarkers

Direct measurement of FRs in clinical samples is challenging because of their high reactivity, short half‐life, and compartmentalized production. Most neonatal studies therefore quantify more stable downstream products, including 8‐hydroxy‐2^′^‐deoxyguanosine as an index of oxidative DNA modification, isoprostanes and lipid hydroperoxides as products of lipid oxidation, advanced oxidation protein products and ortho‐tyrosine as markers of protein oxidation, and 3‐nitrotyrosine as an indicator of protein nitration. Other measurements, such as TOS, total antioxidant capacity, and the OS index, are composite indices and do not identify the reactive species involved, their cellular source, or the affected organ.

Interpretation is further complicated by differences in gestational and postnatal age, biological matrix, sampling time, assay methodology, sample processing and storage, renal and hepatic function, nutrition, transfusions, infection, oxygen exposure, respiratory support, and overall illness severity. Concentrations measured in cord blood or peripheral blood reflect the systemic redox status and may originate from maternal, placental, fetal, or neonatal compartments. They cannot be assumed to represent oxidative injury within the lung, brain, retina, intestine, heart, growth plate, or bone. Similarly, detection of an oxidative modification does not establish whether redox dysregulation contributed to the development of tissue injury or was merely a consequence of the disease severity.

Tissue‐proximal samples, such as tracheal aspirates for lung injury or cerebrospinal fluid for neurological injury, may provide greater anatomical relevance but remain imperfect, are not always safely accessible, and may still contain contributions from multiple cell types. No oxidative or nitrosative biomarker is currently sufficiently tissue‐specific, disease‐specific, standardized, and externally validated to guide routine neonatal diagnosis, prognosis, or treatment.

Greater specificity may be achieved by combining serial rather than isolated measurements of oxidation products with organ‐specific injury or functional markers and detailed clinical phenotyping. Integrated panels incorporating redox biomarkers, inflammatory mediators, metabolomic or proteomic signatures, and tissue‐relevant clinical outcomes may ultimately be more informative than any single systemic marker.

## 5. Conclusions

This review examined the biochemical pathways of free‐radical generation and their role in oxidative damage, with a focus on their involvement in various neonatal pathologies.

Redox biology contributes to physiological signaling and adaptation during fetal and neonatal development, whereas excessive, prolonged, or spatially dysregulated production of reactive oxygen and nitrogen species may amplify cellular and tissue injury. Experimental studies provide mechanistic evidence that redox dysregulation can affect the developing lung, brain, retina, intestine, erythrocytes, cardiovascular system, and skeleton. However, the strength and nature of this evidence differ substantially among organs. Most neonatal clinical studies are observational and measure stable products of oxidative or nitrosative modification in blood, urine, tracheal aspirates, cerebrospinal fluid, or amniotic fluid rather than reactive species directly within the affected tissue. These studies establish associations but generally cannot determine whether redox imbalance is a cause, consequence, or marker of disease severity.

The developing skeleton represents a potentially underestimated target of perinatal redox dysregulation. Cell and animal models indicate that excessive reactive species may impair chondrogenesis, alter osteoblast and osteoclast activity, disrupt collagen and osteogenic signaling, and interfere with skeletal growth. Human evidence remains preliminary. In a longitudinal cohort, cord blood concentrations of 8‐hydroxy‐2^′^‐deoxyguanosine and advanced oxidation protein products were inversely associated with subsequent REMS‐derived skeletal Z‐scores during the first year of life.

OS biomarkers may eventually contribute to risk stratification, but none is currently sufficiently standardized, organ‐specific, or externally validated for routine neonatal decision‐making. Future studies should define gestational‐ and postnatal‐age‐specific reference ranges, standardize biological matrices and sampling times, account for clinical confounders, and determine whether biomarkers add predictive value beyond established clinical variables. For skeletal outcomes, adequately powered longitudinal studies should integrate validated imaging, biochemical markers of bone metabolism, nutrition and medication exposure, growth, and fracture outcomes.

Current neonatal management should continue to emphasize established preventive and supportive measures, including careful oxygen targeting, reduction of avoidable hyperoxia and hypoxia–reoxygenation, human milk feeding, adequate protein and micronutrient provision, appropriate mineral and vitamin D supplementation, infection prevention, and limitation of potentially harmful exposures when clinically feasible. Although antioxidant and redox‐modulating interventions have shown beneficial effects in selected experimental models, the clinical evidence remains insufficient to recommend their routine use for preventing neonatal oxidative injury or impaired bone development. Because reactive species also participate in physiological signaling, vascular regulation, host defense, and tissue development, indiscriminate antioxidant treatment may be ineffective or potentially harmful. Translation into clinical practice will therefore require adequately powered trials evaluating dose, timing, target population, and clinically meaningful long‐term outcomes.

Figure 1 shows redox dysregulation and organ involvement in the newborn. Perinatal exposures, including the physiological oxygen transition at birth, supplemental oxygen, hypoxia–reoxygenation, inflammation, infection, mechanical ventilation, redox‐active iron, and nutritional or pharmacological factors, may modify the production of reactive oxygen and nitrogen species. FRs and non‐radical reactive species exert physiological signaling functions at controlled concentrations, whereas excessive, prolonged, or spatially dysregulated production may cause lipid, protein, and DNA modifications and alter redox‐sensitive pathways. These processes have been associated with injury involving the lung, brain, retina, intestine, erythrocytes, cardiovascular system, and developing skeleton. The biomarkers shown are stable downstream products or composite systemic indices rather than direct measurements of FRs. They should not be considered tissue‐specific, and the arrows indicate biologically plausible or clinically observed associations rather than proven causal relationships.

**Figure 1:**
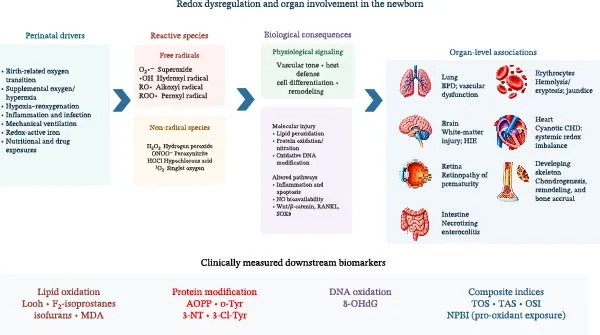
Proposed pathways linking perinatal redox dysregulation to organ injury and neonatal disease. Endogenous and treatment‐related stimuli may disrupt redox homeostasis by increasing reactive oxygen and nitrogen species production, impairing antioxidant defenses, or both. The resulting oxidative and nitrosative modifications may affect lipids, proteins, and nucleic acids and interact with inflammatory and cell‐death pathways. These mechanisms have been implicated, with different levels of preclinical and clinical evidence, in injury to the developing brain, lung, retina, intestine, cardiovascular system, erythrocytes, and skeleton. Biomarkers indicate different components of this process. Abbreviations: AOPP, advanced oxidation protein products; BPD, bronchopulmonary dysplasia; CHD, congenital heart disease; HIE, hypoxic–ischemic encephalopathy; MDA, malondialdehyde; NEC, necrotizing enterocolitis; RNS, reactive nitrogen species; ROP, retinopathy of prematurity; ROS, reactive oxygen species; 8‐OHdG, 8‐hydroxy‐2^′^‐deoxyguanosine.

**Nomenclature**
FR: Free radicals
ROS: Reactive oxygen species
RNS: Reactive nitrogen species
O_2_•^−^: Supsuperoxide
•OH: Hydroxyl
HO_2_•: Hydroperoxyl
RO•: Alkoxyl
ROO•: Peroxyl
H_2_O_2_: Hydrogen peroxide
HOCl: Hypochlorous acid
^1^O_2_: Singlet oxygen
O_3_: Ozone
NO•: Nitric oxide
NO_2_•: Nitrogen dioxide
ONOO^−^: Peroxynitrite
HNO_2_: Nitrous acid
N_2_O_3_: Dinitrogen trioxide
nNOS/NOS1: Neuronal NOS
eNOS/NOS3: Endothelial NOS
iNOS/NOS2: Inducible NOS
MS: Mass spectrometry
LC‐MS: Liquid mass spectrometry
OS: Oxidative stress
RDS: Respiratory distress syndrome
BPD: Bronchopulmonary dysplasia
8‐OHdG: 8‐hydroxydeoxyguanosine
LTE_4_: Leukotriene E_4_
WMI: White matter injury
HIE: Hypoxic–ischemic encephalopathy
ROP: Retinopathy of prematurity
TOS: Total oxidant status
MDA: Malondialdehyde
TAS: Total antioxidant status
NEC: Necrotizing enterocolitis
CHD: Congenital heart disease
3NO_2_‐Tyr: 3‐nitro‐tyrosine
3Cl‐Tyr: ‐chlorotyrosine
HO‐1: Heme oxygenase‐1.

## Author Contributions

Conceptualization: Serafina Perrone. Methodology: Laura Cannavò, Virginia Beretta, and Serafina Perrone. Writing – original draft preparation: Serafina Perrone, Laura Cannavò, and Virginia Beretta. Writing – review and editing: Serafina Perrone, Laura Cannavò, Virginia Beretta, Chiara Petrolini, Vincenzo Raitano, Elena Scarpa, Valentina Dell’Orto, Silvia Carloni, and Maria Cristina Albertini.

## Funding

PNNR‐MAD‐2022‐12376819 project funded under the National Recovery and Resilience Plan (NRRP) (M6/C2_CALL 2022 Italian Ministry of Health, funded by the European Union—NextGenerationEU). Open access publishing facilitated by Universita degli Studi di Parma, as part of the Wiley ‐ CRUI‐CARE agreement.

## Disclosure

All authors have read and agreed to the published version of the manuscript.

## Conflicts of Interest

The authors declare no conflicts of interest.

## Data Availability

Data sharing is not applicable to this article as no datasets were generated or analyzed during the current study.
